# Working at the interface of physics and biology: An early career researcher perspective

**DOI:** 10.1016/j.isci.2022.105615

**Published:** 2022-11-25

**Authors:** Claire Dilliway, Oliver Dyer, Elena Mandrou, Daniel Mitchell, Govind Menon, Hugh Sparks, Valentin Kapitany, Alex Payne-Dwyer

**Affiliations:** 1 Department of Earth Sciences and Engineering, Imperial College London, London, UK; 2 Department of Physics, Warwick University, Coventry, UK; 3 Cancer Research UK Beatson Institute, University of Glasgow, Institute of Cancer Sciences, Glasgow, Scotland, UK; 4 Henry Wellcome Building for Biocatalysis, University of Exeter, Exeter, UK; 5 Department of Computational and Systems Biology, John Innes Centre, Norwich, UK; 6 Photonics Group, Department of Physics, Imperial College London, London, UK; 7 University of Glasgow, School of Physics & Astronomy, Glasgow, Scotland, UK; 8 School of Physics, Engineering and Technology, University of York, York, UK

## Abstract

We are a network of Early Career Researchers (ECRs) and a Project Manager who are working on UKRI’s “Physics of Life” grants which aim to merge ideas and techniques predominantly used in physics and apply them to biological questions. We have been collaborating since early 2021 to share research, experiences, and provide peer to peer support.

Interdisciplinary projects are known for presenting challenges, bringing together disparate subjects and people with not only different knowledge bases, methods, and equipment but also varying ways of working and common languages. This has been the subject of commentary by researchers and funders from a management perspective, and we wanted to add to this discourse, using our experience to share the lessons and challenges we have encountered, from an ECR perspective.

**Figure:**
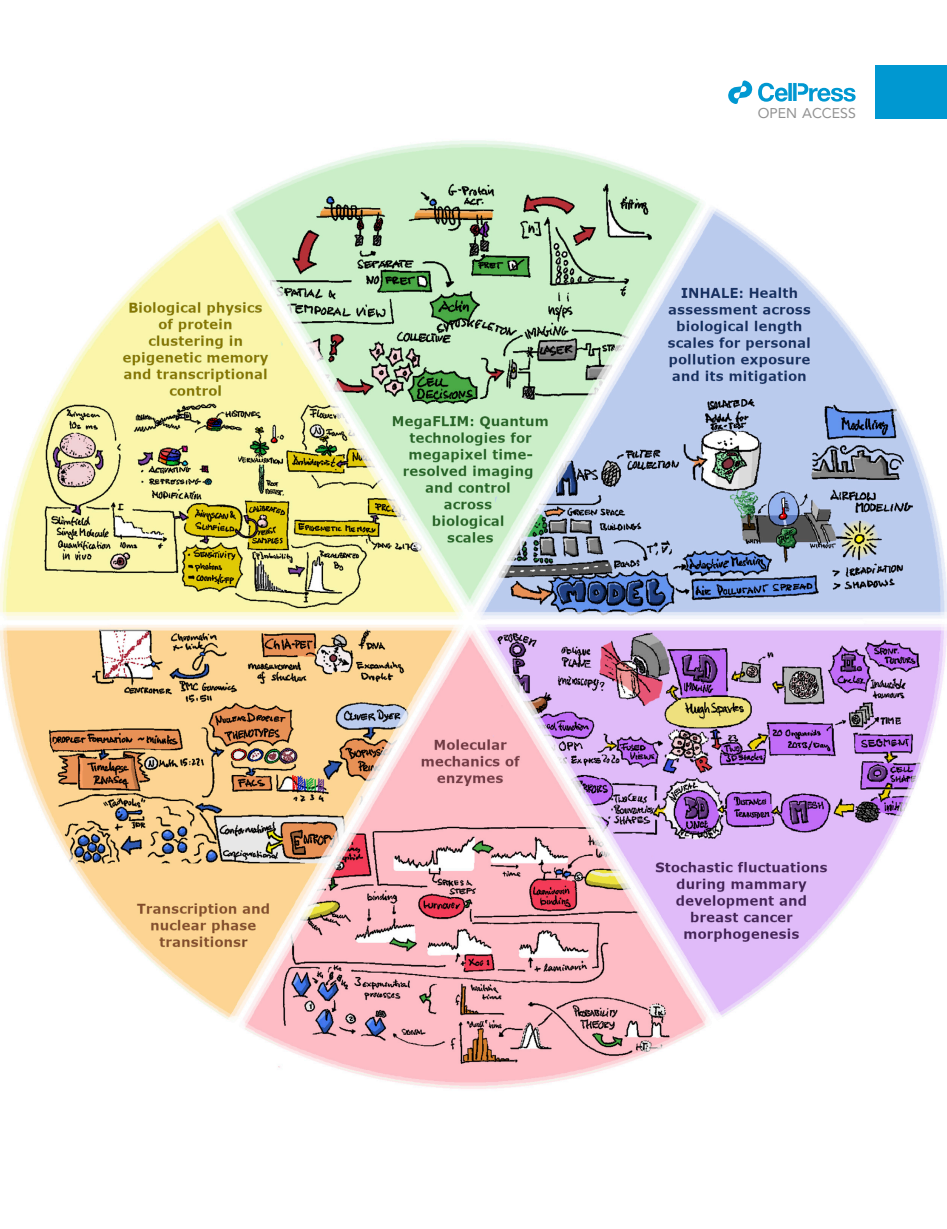
Physics of Life Projects Collage: Produced from sketch notes taken by Dr. Mathis Riehle, University of Glasgow, at the PoL ECR workshop in July 2021. Originals: https://www.physicsoflife.org.uk/physics-of-life-ecr-workshop.html.

Time losses can happen before projects even start.

papers that are equally balanced between the subject specialisms tend to be cited less than those which use techniques from a range of areas to tackle a profound question on one specific area,^1^ showing that truly interdisciplinary science is still in its infancy and requires time and resources to expand upon.

To create time for these team building activities and account for delays in recruitment we would advise that this is taken into consideration for future calls by incorporating an inception period.

the experience of working closely with other disciplines can be very rewarding and give rise to opportunities which would otherwise have been inaccessible.

## Main text

### “Physics of Life” projects as case studies

To illustrate how researchers from different disciplines have formed consortia to bring new insight and methods to bear on traditionally biological questions, and to introduce ourselves, we use our projects as case studies below.

#### Molecular mechanics of enzymes (University of Exeter)

Daniel Mitchell is a postdoctoral research associate and biochemist working on the molecular mechanics of enzymes project. This project investigates single-molecule enzyme turnover, something that has so far remained elusive. To achieve this, we employed the phenomenon of “whispering gallery modes”. This involves exploiting the precise measurement of the optical resonance frequency of light to detect very small changes in enzyme conformation. Light can be passed into a microsphere and trapped due to total internal reflection. This results in self-interference creating an optical resonance, with the waves extending slightly from the sphere via an evanescent field. By attaching a gold nanoparticle to the sphere, the light field can be strongly localized to enable detection of very small changes such as single enzyme turnover.^2^^,^^3^ More information can be found here: www.exeter.ac.uk/research/livingsystems/research/mmi.

#### INHALE: Health assessment across biological length scales for personal pollution exposure and its mitigation (Imperial College London)

Claire Dilliway is the Program Manager working on INHALE, among others, at Imperial College London. INHALE assesses the impact of air pollution on personal health in urban environments. Health responses to pollution from participants in our clinical study are gathered using a combination of medical examinations/samples and personal and stationary fine particle exposure monitors. Using particulate matter collected from various microenvironments around West London, we examine the biophysical components of pollutants that determine their potential to cause cell/tissue damage also via *in vitro* studies.^4^ These data are integrated to develop pollution-health impact models from the cell, lung, and person, up to the neighborhood scale, investigating the effect of various intervention scenarios (e.g., roadside hedges or medication for asthmatics). Read more: www.imperial.ac.uk/inhale.

#### MegaFLIM: Quantum technologies for megapixel time-resolved imaging and control across biological scales (CRUK Beatson Institute & University of Glasgow)

Elena Mandrou (CRUK Beatson Institute) and Valentin Kapitany (University of Glasgow) are PhD students involved in the MegaFLIM project. The MegaFLIM project aims to develop and employ advanced computational and quantitative microscopy techniques to image signaling in cancer cell clusters much like those found in patients. Chemical signal integration and response has been studied extensively in single cells, but less is known about how cell collectives dynamically signal across multicellular scales. We can measure these behaviors via fluorescence lifetime imaging microscopy (FLIM) by using cancer cells expressing G-protein biosensors that report on Förster resonance energy transfer (FRET) states. A statistics-based approach is used to estimate FRET efficiency with high spatial fidelity, by combining time-resolved single-photon quantum detectors with high-resolution cameras.^5^^,^^6^

#### Transcription and nuclear phase transitions (University of Warwick)

Oliver Dyer is a postdoctoral researcher and computational physicist at the University of Warwick, whose project investigates the role of liquid-liquid phase transitions (LLPTs) in mammalian cell nuclei, especially with regards to the machinery of transcription. We are using an *in vivo* LLPT induction system along with next-generation sequencing techniques and imaging approaches to generate a clearer profile about transcriptional phenomena, including initiation, promoter-proximal pausing, and early termination.

To characterize the physical properties of LLPT-induced droplets, we use experimental techniques such as fluorescence recovery after photo bleaching and single particle tracking alongside simulations modeling nuclear constituents in terms of their entropic properties only, revealing surfactant-like behavior of key proteins.^7^ Read more: www.warwick.ac.uk/fac/cross_fac/transcription/

#### Biological physics of protein clustering in epigenetic memory and transcriptional control (John Innes Center and University of York)

Govind Menon (John Innes Center) and Alex Payne-Dwyer (University of York) are postdoctoral researchers whose project investigates a widely conserved epigenetic mechanism—silencing by the Polycomb repressive pathway—a form of gene regulation underlying a broad spectrum of developmental processes in multicellular organisms from plants to humans. This project uses the plant gene *FLC (Flowering Locus C)* as its core model system,^8^ and examines the functional importance of protein clustering for controlling transcription and epigenetic memory at this gene. The project combines quantitative biophysical approaches—super-resolution imaging techniques in live plant tissues^9^—with an existing long-term collaboration in plant molecular biology and theoretical modeling.

#### Stochastic fluctuations during mammary development and breast cancer morphogenesis (Francis Crick Institute and Imperial College London)

Hugh Sparks is a postdoctoral researcher at Imperial College London whose project bridges the gap between organoid biology and physics of non-equilibrium systems to study fundamental cellular processes underlying the development of normal and breast cancer mouse organoids. To measure the three-dimensional self-organization of mammary organoid structures over time, experimental physicists developed a new folded dual-view oblique plane microscopy (OPM) technique termed dOPM.^10^ To quantify the dOPM datasets, theoretical physicists developed computational methods to automatically segment and track individual cells within each organoid.^11^ Finally, to help with the interpretation of segmentation analysis results, a physical model of organoids based on interacting active surfaces was developed.

### Bringing the experience together

#### How did the interdisciplinary workshop come about?

During a Physics of Life grant holders meeting, hosted by EPSRC in January 2021, there was a discussion about how ECR career development, as the next generation of truly interdisciplinary scientists, could be supported. To this end, project leaders were asked to nominate representatives (the authors) to organize a workshop or similar.

Initially, there were the usual challenges of coordination and getting buy-in from all projects. Firstly, everyone was so busy, but also, (and this was a recurring challenge) because the projects were so different in nature. One group felt that their project was so far removed from the others and so did not participate, for example.

We had little detail on what the intended outcomes of the workshop were intended to be. Furthermore, with our disparate projects, it was initially difficult to tie down what the aims should be, and, therefore, how best to structure the workshop and achieve a meaningful outcome for all involved.

#### What were the aims?

Ultimately, we decided to provide a forum for ECRs to present their work. Rather than covering the science behind each project in detail, there was a particular focus for the speakers to truly take stock of what their projects had achieved and how to communicate that to the wider scientific community. This cross-discipline communication was felt to be an important skill for scientists working across the physics of life spectrum, while additionally providing a different perspective from non-experts working in a similar area, and potentially leading to new ideas. We also wanted to draw out the ECR experience of working on interdisciplinary projects, to share lessons learned, and identify areas of possible collaboration across the projects. Using that as our basis, and with help from Karis Baker at PolNet, we planned how best to structure the workshop.

#### What were the challenges?

Once the aims of the workshop had been agreed, each of the representatives from the projects had to relay these aims back to their teams and get agreement from their ECRs to present their work for a general audience in a total of 45 min per project. This had all the challenges (and benefits) associated with bringing together scientists of different specialisms from across each project to create a combined talk that could be understood by a general scientific audience, using less specialized language and explaining some key concepts. We additionally wanted to encourage other physics of life early career scientists from outside of the projects presenting to attend so that they could learn from our experience, give another perspective, and encourage new collaborations.

Of course, we were also in the midst of the COVID-19 pandemic, and the associated restrictions which we had to account for in our planning.

This was a valuable experience for all of us and, for some, a first experience of organizing such an event. You can see the workshop summary, program, sketch notes, and recordings of the talks here: https://www.physicsoflife.org.uk/physics-of-life-ecr-workshop.html.

### Common challenges and solutions for interdisciplinary projects

#### What were the lessons learned?

From the workshop and the discussions among our network (the authors), two common themes emerged—that of time and the unconventional route to publication outputs. Without permanent positions, ECRs need to demonstrate outputs from within the time frame of the project when applying for their next role. It is therefore crucial to be aware of the additional time investments in interdisciplinary projects. These are present from the very start of the project, when researchers need to familiarize themselves not only with a new topic or application, but often an entirely new discipline with its own ways of communicating ideas.

Time losses can happen *before* projects even start. The process between funding call and project start in our case was rushed, leading to many ECR posts being filled months *after* the project started. While we acknowledge such processes are influenced by many factors, it was an avoidable and unfortunate set of circumstances adding to the considerable existing challenges in projects of this nature.

Adapting techniques and equipment to new types of analyses can also be challenging and time-consuming. For example, applying established techniques for imaging or processing one type of biological sample (e.g., bacterial cells) to a completely different one (e.g., intact plant tissue). This may require adjustments to be made in the equipment, sample preparation protocol, operating procedures, and conditions, as well as identifying new experimental controls. Furthermore, ensuring data are portable across the various analysis tools used in different disciplines adds another layer of work. This would ideally be discussed and agreed beforehand, but is sometimes dealt with retrospectively.

Collectively, these factors hinder ECRs’ abilities to generate recognized project outputs before the end of the funding period, even if their work has been productive. In addition, papers that are equally balanced between the subject specialisms tend to be cited less than those which use techniques from a range of areas to tackle a profound question on one specific area,^1^ showing that truly interdisciplinary science is still in its infancy and requires time and resources to expand upon. This is further compounded for ECRs by interdisciplinary papers tending to be cited less in the short term,^12^ despite there being a positive correlation between the number of subject specialisms the paper touches upon and number of citations in the long term.^13^

The preparation of interdisciplinary journal articles—often led by ECRs—has similar challenges as the initial stages of the project, with authors needing to scour disconnected literature and meet expectations from different areas. Accounting for this can multiply the burden of planning and work required to be accepted for publication. Furthermore, choosing the right journal to publish a multidisciplinary article is not always straightforward.

The unique technical and communication skills gained by interdisciplinary ECRs are now well recognized by learned societies and increasingly by funding bodies including those in UKRI and charities. Yet the path to translating this into a successful career in science remains narrower and riskier than for our single-discipline colleagues. However, there is room for some positivity; more interdisciplinary departments are being established in research institutes, such as the Advanced Research Center at the University of Glasgow and the Living Systems Institute at the University of Exeter.

#### How do we build interdisciplinary teams?

As we have previously noted, projects led by teams composed of scientists from disparate disciplines are known to be challenging. In most cases, individuals in a team do not possess both practical and theoretical knowledge of multiple subjects. All authors therefore found that working closely together and fostering sustained knowledge exchange has been key to integrating physics and biology successfully. This is particularly applicable to ECRs who are often at the forefront of the practical implementation of experiments and work toward the goal of bringing new solutions to existing problems. The table below identifies some of the main ways this can be achieved.

One additional challenge during these projects was that COVID restrictions were in place which hindered many of the activities listed in Table 1. However, they also presented an opportunity to adapt. For example, one author learned how to perform computational chemistry experiments when lab access was limited.

**Table 1:** Ways to build effective interdisciplinary teams

What	Why	How
Knowledge exchange	Creating a shared language between project members	• Circulating the project proposal between all members (ECRs often do not see this as they are employed when the grant is already running) • Giving general scientific presentations from different areas of the project to familiarize members with different methods and disciplines • Shadowing colleagues from other disciplines in their laboratories
Holding regular meetings	For planning, meeting targets, and adapting to new information and problem solving	• Planning from the beginning how the work will be integrated to ensure that the project will be more than the sum of its parts • General meetings across project members to share progress updates and to solve problems • Meetings with smaller more focused groups to solve technical problems • Open-ended discussions to inspire new ideas and to guide the design of unexpected experiments and analysis
Connecting and communicating online	To facilitate efficient communication between project members and labs in different locations	• Cloud storage • Online communications tools • Access to relevant journals • Sharing and developing software collaboratively
Informal events	To strengthen collaborations and celebrate successes	• Online or in-person socials • Fun team-building activities

While there are many benefits to employing the approaches in Table 1, it is important to highlight that time investment is required. To create time for these team-building activities and account for delays in recruitment, we would advise that this is taken into consideration for future calls by incorporating an inception period. This would facilitate the establishment of deep cross-discipline understanding and detailed planning to address the challenges highlighted here to maximize project success.

#### How can this knowledge be extended to other interdisciplinary projects?

Interdisciplinary work is becoming fundamental for gaining new insights and tackling important questions and can only be expected to grow and become more common as a way of working. We hope that the benefits and challenges we have identified from our experience of projects sitting at the interface of physics and biology, with a focus on the ECR perspective, can be helpful to those working on or considering projects which are interdisciplinary in nature. ECRs might wish to note the time investment required in working on projects of this kind and potential delays and challenges in publishing. While these can be frustrating, the experience of working closely with other disciplines can be very rewarding and give rise to opportunities which would otherwise have been inaccessible. To meet these challenges, we have discussed a range of ways in which an interdisciplinary project could be undertaken using our combined experience and hope this serves as a useful resource for future interdisciplinary projects. Funders may take particular note of the recommendation to incorporate inception periods into these kinds of complex interdisciplinary projects.
